# Estimated health benefits, costs and cost-effectiveness of eliminating dietary industrial trans fatty acids in Kenya: cost-effectiveness analysis

**DOI:** 10.1136/bmjgh-2023-012692

**Published:** 2023-10-17

**Authors:** Matti Marklund, Leopold N Aminde, Mary Njeri Wanjau, Liping Huang, Celine Awuor, Lindsay Steele, Laura K Cobb, J Lennert Veerman, Jason HY Wu

**Affiliations:** 1 Department of Public Health and Caring Sciences, Uppsala University, Uppsala, Sweden; 2 The George Institute for Global Health, University of New South Wales, Sydney, New South Wales, Australia; 3 Department of Epidemiology, Johns Hopkins Bloomberg School of Public Health, Baltimore, MD, USA; 4 Public Health and Economics Modelling Group, School of Medicine and Dentistry, Griffith University, Gold Coast, Queensland, Australia; 5 International Institute for Legislative Affairs, Nairobi, Kenya; 6 Resolve to Save Lives, New York, New York, USA; 7 School of Population Health, University of New South Wales, Sydney, New South Wales, Australia

**Keywords:** Health policy, Public Health, Nutrition, Cardiovascular disease

## Abstract

**Objectives:**

To model the potential health gains and cost-effectiveness of a mandatory limit of industrial trans fatty acids (iTFA) in Kenyan foods.

**Design:**

Multiple cohort proportional multistate life table model, incorporating existing data from the Global Burden of Disease study, pooled analyses of observational studies and peer-reviewed evidence of healthcare and policy implementation costs.

**Setting:**

Kenya.

**Participants:**

Adults aged ≥20 years at baseline (n=50 million).

**Intervention:**

A mandatory iTFA limit (≤2% of all fats) in the Kenyan food supply compared with a base case scenario of maintaining current trans fat intake.

**Main outcome measures:**

Averted ischaemic heart disease (IHD) events and deaths, health-adjusted life years; healthcare costs; policy implementation costs; net costs; and incremental cost-effectiveness ratio.

**Results:**

Over the first 10 years, the intervention was estimated to prevent ~1900 (95% uncertainty interval (UI): 1714; 2148) IHD deaths and ~17 000 (95% UI: 15 475; 19 551) IHD events, and to save ~US$50 million (95% UI: 44; 56). The corresponding estimates over the lifespan of the model population were ~49 000 (95% UI: 43 775; 55 326) IHD deaths prevented, ~113 000 (95% UI: 100 104; 127 969) IHD events prevented and some ~US$300 million (256; 331) saved. Policy implementation costs were estimated as ~US$9 million over the first 10 years and ~US$20 million over the population lifetime. The intervention was estimated to be cost saving regardless of the time horizon. Findings were robust across multiple sensitivity analyses.

**Conclusions:**

Findings support policy action for a mandatory iTFA limit as a cost-saving strategy to avert IHD events and deaths in Kenya.

**WHAT IS ALREADY KNOWN ON THIS TOPIC**

- Intake of trans fatty acids is a well-established risk factor for ischaemic heart disease, and although implementation of policies aiming to eliminate industrial trans fatty acids is increasing around the world, little progress has been made in most African countries including Kenya.
- Estimates of the potential health impacts and cost-effectiveness of a best practice policy to eliminate industrial trans fatty acids could inform policymakers in Kenya and other African countries.

**WHAT THIS STUDY ADDS**

- We estimated that a mandatory limit of industrial trans fatty acids in the food supply could be a cost-saving strategy to reduce the increasing burden of ischaemic heart disease in Kenya.
- For instance, within the first 10 years, the policy could prevent around 17 000 heart disease events.
- The healthcare cost savings due to averted heart disease were estimated to surpass the policy implementation costs for government and industry by severalfold.
- Our findings were robust to a wide range of alternative model inputs and assumptions.

**HOW THIS STUDY MIGHT AFFECT RESEARCH, PRACTICE OR POLICY**

- Our findings support the prompt implementation of a mandatory limit of industrial trans fatty acids in the foods in Kenya as a tool to promote diet and health and save lives.
- In addition to these quantitative estimates potentially helping to inform policymakers and advocate development of a trans fat policy in Kenya, our modelling framework can also be adapted to understand the health and economic impact of other food policies in Kenya and other African countries.

## Background

The burden of ischaemic heart disease (IHD) is rapidly increasing in African countries, including in Kenya, where the number of annual IHD deaths has increased more than threefold since 1990.^1^ Intake of trans fatty acids (TFAs), a group of unsaturated fatty acids with one or more double bonds in the trans configuration, is a well-known dietary risk factor of IHD. TFA causes dyslipidaemia and other cardiometabolic dysregulations.^2^ Across population-based observational studies, every 2% increase in total energy intake from TFAs increased IHD risk by 23%.^3^ TFAs occur naturally at low levels in meat and milk from ruminants, but in most countries intakes are predominantly driven by the use of industrially made partially hydrogenated vegetable oils in processed foods.^4^

Given the adverse effects of TFAs, WHO has prioritised industrial TFA (iTFA) elimination, and recommends limiting TFA intake to less than 1 energy percentage (%E). In 2018, WHO called for the global elimination of iTFA by 2023, and released the REPLACE action package with best practice policy guidance for countries.^5^ Many countries have implemented strategies to reduce industrial-derived TFA (iTFA) in the food supply.^6 7^ These policies range from voluntary reformulation, mandatory labelling, to mandatory limits on iTFA or complete bans on partially hydrogenated oils (the primary source of iTFA).^8^ For example, iTFA content in foods must be ≤2% of total fat in Denmark^9^ and in 2015, the US Food and Drug Administration determined that partially hydrogenated oils are no longer recognised as safe for use in human food, effectively banning their use.^10^

Kenya is considering policy options to regulate iTFA.^11^ However, there is currently no limit (voluntary or mandatory) of iTFA content in foods, fats and oils. To further inform regulations related to iTFA in Kenya, we conducted a modelling study that estimated the health impact and cost-effectiveness of a best practice mandatory limit on iTFA content (≤2% of all fats) in foods, fats and oils in Kenya, taking into account policy costs, reduced IHD burden and healthcare expenditures. We hypothesised that a limit of iTFA in the food supply would be a cost-effective measure to reduce IHD burden in Kenya.

## Methods

### Study design

We used a multiple cohort proportional multistate life table (Markov) model to estimate the impact on health outcomes and related costs of an iTFA limit (≤2% of all fats) for the Kenyan food supply (online supplemental figure S1). The limit is in line with the WHO-recommended best practice policy for iTFA elimination and applies to iTFA in all foods and ingredients (eg, partially hydrogenated vegetable oils). We adapted a previously developed model for Australia,^12^ which was constructed to calculate IHD-related outcomes and total healthcare costs resulting from the intervention.^13 14^ The life table method transmits changes in iTFA intake to IHD-related morbidity and mortality in the modelled population. In all analyses, the adult Kenyan population (≥20 years) was modelled in 5-year male and female cohorts, simulating each cohort until all individuals died or reached 100 years of age. Outcomes were compared between a reference population with TFA intake of the Kenyan population before the intervention and an intervention population with identical characteristics but lower TFA intake as a result of eliminating iTFAs from the food supply. The differences in health outcomes between reference and intervention populations were expressed in IHD incidence and deaths, life years and health-adjusted life years (HALYs). Results were reported for time horizons of 5 years, 10 years and lifespan (ie, the time from policy implementation until all individuals died or reached 100 years of age). We used an ‘extended’ health sector perspective that included implementation costs for government and industry, as these are directly related to the intervention. Costs were inflated to 2019 values. In line with the recommendations of the first and second panels on cost-effectiveness in health and medicine, we used a 3% discount rate in the main analysis.^15 16^ Key inputs and assumptions are presented in table 1.

10.1136/bmjgh-2023-012692.supp1 Supplementary data

**Table 1:** Key input data and assumptions

Input	Stratification	Values	Source	Note
Preintervention TFA intake, %E	Age, sex	Online supplemental table S1	2019 Global Burden of Disease (GBD) study	For each model iteration, random draws from age-sex-specific lognormal TFA distributions were made.
Postintervention TFA intake, %E	n/a	0 (primary analysis) Mean±SD: 0.1±0.01 (sensitivity analysis)	2018 Global Dietary Database (estimates of dairy intake)	The intervention was assumed to virtually eliminate TFA intake in Kenya, given the minimal intake of naturally occurring (ruminant sources of) TFA in Kenya compared with countries like Australia, UK and Denmark. In a sensitivity analysis, we explored the impact of a low TFA intake from ruminant sources or suboptimal compliance to limit imposed on iTFA.
Theoretical minimum risk distribution of TFA intake, %E	n/a	0 (primary analysis) Mean±SD: 0.1±0.01 (sensitivity analysis)	Marklund *et al*^12^	The theoretical minimum risk distribution of TFA intake was assumed to equal the intake of naturally occurring TFA.
Population size	Age, sex	Online supplemental table S2	2019 GBD study	
Mortality rate	Age, sex	Online supplemental table S2	2019 GBD study	
IHD incidence, prevalence and case fatality rates	Age, sex	Online supplemental table S3	2019 GBD study	
RR for IHD per 2%E from TFA	Age	Online supplemental table S4	Afshin *et al*^21^	For each model iteration, random draws from age-specific lognormal RR distributions were made.
Disability weights	Age, sex	Online supplemental tables S5 and S6	2019 GBD study	
Healthcare costs	Age, sex	Online supplemental table S7 Acute IHD event: $6283 (men)/$6083 (women) Annual cost per prevalent case: $336	Subramanian *et al*^23^ Gaziano *et al*^28^	For incident IHD, we pooled estimates for myocardial infarction ($1996 per event), angina ($1237 per event) and cardiac arrest secondary to hypertension ($1026 per event) weighted for their relative contribution to total acute IHD events. The annual cost per prevalent IHD case was derived from the costs of chronic secondary prevention for all coronary heart disease states. For each model iteration, random draws from age-sex-specific normal distributions of costs for IHD incidence and prevalence as well as non-IHD healthcare costs, assuming SD equals to 20% of central estimates.
Government policy implementation costs	n/a	Online supplemental table S8	Ngalesoni *et al*^32^	Five categories of costs were considered: strategy development and evaluation (including development and legislation of laws); human resources (for programme management and law enforcement); promotion and media advocacy; office rent, equipment and supplies; and administration. For each model iteration, random draws from normal distributions of costs for each category, assuming SD equals to 20% of central estimates.
Industry reformulation	n/a	Online supplemental table S8	Marklund *et al*^12^	Reformulation costs were calculated using equivalent US$ costs from UK estimates (£25 000 per product)^5^ multiplied by the number of products in the Kenyan food supply potentially containing iTFA (primary model: n=99; sensitivity analysis: n=198). Annual cost to industry equalling 1% of the initial reformulation cost was assumed. For each model iteration, random draws from normal distributions of initial and annual reformulation costs, assuming SD equals to 20% of central estimates.

%E, energy percentage; IHD, ischaemic heart disease; iTFA, industrial TFA; n/a, not available/applicable; RR, relative risk; TFA, trans fatty acid.

### Data sources

#### Intake of TFA in Kenya

Baseline intake (mean and SD) of TFA, expressed as %E, per age and sex group (n=30 groups in total) was derived from the 2019 Global Burden of Disease (GBD) study (online supplemental table S1). We modelled the effect of a mandatory limit of iTFA to ≤2% of all fats in food products. For the primary analysis we assumed such a policy will eliminate TFA intake of all sex-age groups, that is, postintervention mean intake=0%E (table 1). The postintervention intake level was set at 0%E because (1) a similar policy eliminated iTFA from processed foods in Denmark,^17^ and (2) the likely negligible intake of non-industrial-derived TFAs (ie, from meat and dairy from ruminant animals) in Kenya.^14 18^

#### Health outcomes

We used age-specific and sex-specific IHD incidence, prevalence, mortality rates for Kenya and the 2019 population data estimates from the GBD 2019 study to populate our model (online supplemental tables S2 and S3).^1 19^ The GBD study makes available to researchers the estimated GBD in 204 countries and territories. The GBD study uses various primary data sources for each country. For Kenya, their main primary sources of data included the Demographic and Health Surveys, Kenya Multiple Indicator Cluster Surveys, Kenya World Health Surveys, Kenya STEP Skills Measurement Household Surveys, Kenya STEPS Noncommunicable Disease Risk Factors Survey 2015 and Kenya Population and Housing Census. A detailed list of primary data sources is available through the GBD 2019 sources.^1^

We used DisMod II software^20^ to enforce internal consistency in the IHD epidemiological estimates obtained from GBD 2019 study while deriving IHD case fatality rates that are not provided in the GBD data (online supplemental methods and online supplemental figure S1). The software uses a set of differential equations that exploit the causal relation in a typical disease process to estimate absent epidemiological parameters while maintaining stability in the overall disease epidemiology.^20^ Estimates of IHD incidence, prevalence and case fatality per sex and year of age were generated using DisMod II (online supplemental table S3). Age-specific relative risks (RR) of TFA intake and IHD were based on meta-analyses of findings from prospective cohort studies (online supplemental table S4).^21^ We calculated disability weights using disease-specific prevalence and years lived with disability (YLDs) estimates from the 2019 GBD study (online supplemental appendix and online supplemental tables S5 and S6).^1 22^ Our model included adults aged 20–100 years and 50% of the total model population were women (online supplemental table S2). Children and adolescents (age <20 years) were not included in the model, given the low IHD burden and the lack of well-established RRs of TFA intake and IHD in that age group.

10.1136/bmjgh-2023-012692.supp2 Supplementary data

#### Healthcare and policy costs

We conducted a literature search to identify the best estimates of the total health expenditure and IHD healthcare costs in Kenya for use in our modelling study. For costs per incident IHD case, we used annual costs of acute myocardial infarction, angina and cardiac arrests (heart failure due to hypertension) from a study that quantified the cost of non-communicable diseases (NCD) in public and private sectors in Kenya.^23^ These conditions were considered the acute presentations of IHD, and thus were linked to IHD incidence in the model. In line with previous research, we assumed that cardiac arrest represented 10% of all acute IHD events, myocardial infarction 20% (females) to 35% (males) and angina the remaining acute IHD events (ie, 70% for females and 55% for men).^24–26^ While utilisation of private healthcare providers (where cost of IHD-related care is higher) is considerable in Kenya,^27^ we conservatively used cost estimates from Kenya’s public healthcare sector. For the annual cost per prevalent IHD case, we used the costs of chronic secondary prevention for all coronary heart disease states as calculated by Gaziano and colleagues, based on data from South Africa.^28^ We assumed the SD equals 20% of the point estimates. The total healthcare expenditure in Kenya was from the 2020 WHO Global Health Expenditure Database that published 2018 costs.^29^ Information published in the 2013 Kenya Household Health Expenditure and Utilisation Survey^30^ was used to apportion Kenya’s total health expenditure on IHD to males and females and across age.

In addition to costs for the IHD in the model, overall healthcare costs for all other health conditions are also included. This is necessary because as interventions prolong life, additional health expenditure is expected in those added years of life.^31^ To derive the costs of all other diseases per person, we subtracted the costs of incident and prevalent IHD for each age group from the total health expenditure in the respective age-sex group. Total, IHD-related and other healthcare costs are presented in online supplemental table S7.

Due to lack of available data on the likely cost of implementing a mandatory iTFA limit for foods in Kenya, we based our estimates on a robust costing study conducted as part of a cost-effectiveness analysis of tobacco control policies in Tanzania.^32^ This study was guided by the tobacco control interventions costing segment contained in the WHO NCD costing tool.^33^ Authors used country-specific data to establish the total costs for the tobacco control policy in Tanzania (reported in US$). We used these estimates based on several assumptions. Tanzania and Kenya are neighbouring countries in East Africa with similar population sizes, and both are lower middle income countries and are thus likely to share certain broad similarities in economy and living standards. Second, we considered the tobacco control/smoking ban policies and the iTFA ban from the food supply to be somewhat similar upstream health-promoting interventions that would require broadly similar implementation resources. The Tanzania costing study included five cost components: strategy development and evaluation (including development and legislation of laws); human resources (for programme management and law enforcement); promotion and media advocacy; office rent, equipment and supplies; and administration. While the Tanzanian costing study did not report costs after 5 years, we assumed costs for human resources and policy administration to remain constant after year 5 (table 1 and online supplemental table S8).

Reformulation costs for industry were calculated using equivalent US$ costs from UK estimates (£25 000 per product)^34^ multiplied by the number of products in the Kenyan market potentially containing iTFA (table 1 and online supplemental table S8). We estimated this number using a large nutrition composition database, the 2018 Kenyan FoodSwitch database (which includes products collected over 2 months in 2018 in five major supermarket chains in Nairobi).^35^ Products were identified that contained any terms indicative of iTFA in the ingredient list (ie, ‘partially hydrogenated fat’, ‘hydrogenated vegetable oil’ or ‘hydrogenated’), as previously described.^36^ Of a total 5668 unique packaged food products with ingredient information included in the analysis, 99 products (1.7%) contained specific ingredients indicative of iTFA.^36^ In line with previous modelling studies,^34 37^ we assumed an ongoing annual industry cost equalling 1% of the initial reformulation cost to conservatively account for reduced industry profits.

We inflated all costs (ie, both healthcare and implementation costs) to 2019 (ie, the model base year) using the US Bureau of Labor Statistics’ Consumer Price Index.^38^ The costs were estimated in US$ and converted to local currency (Ksh) using the average exchange rate of 1 July 2019 (US$1=Ksh103).

### Statistical analysis

#### Estimation of health benefits and cost-effectiveness

The reference and intervention TFA intakes and the RR of IHD per %E of TFA intake were used to calculate the potential impact fraction (PIF) for estimation of the proportional change in IHD incidence due to the elimination of iTFAs (equation 1). Barendregt’s continuous ‘distribution shift’ PIF method was used.^39^

(1) $${PIF}_{as}=\frac{\int_{x=0}^{m}RR_{a}\left(x\right)P_{as}\left(x\right)dx-\int_{x=0}^{m}RR_{a}\left(x\right)P_{as}^{\prime }\left(x\right)dx}{\int_{x=0}^{m}RR_{a}\left(x\right)P_{as}\left(x\right)dx}$$

The PIF_as_ is the potential impact fraction for age group a and sex s, RR_a_(x) is the relative risk as a function of the exposure x (ie, TFA intake), P_as_(x) is the reference TFA intake distribution and P’_as_(x) is the intervention TFA intake distribution. In the primary analysis, we assumed the iTFA intake to be virtually eliminated, and given the negligible intake of naturally occurring TFA, the total TFA intake in Kenya was consequently assumed to be eliminated as well. Hence, for the primary model, a simplification of the PIF equation (ie, a population attributable fraction equation) was used (equation 2).

(2) $${PIF}_{as}=\frac{\int_{x=0}^{m}RR_{a}\left(x\right)P_{as}\left(x\right)dx-1}{\int_{x=0}^{m}RR_{a}\left(x\right)P_{as}\left(x\right)dx}$$

The PIF was used to calculate the effect on IHD incidence due to the reduction in TFA intake (equation 3).

(3) I`=I(1-PIF)

I is the IHD incidence in the reference population, I’ is the IHD incidence in the intervention population and PIF is the potential impact fraction. The estimated incidence rates were used in the disease Markov model to calculate reference and intervention IHD prevalence and mortality. The changes in IHD mortality rate then feed into the life table to alter the overall mortality rates and recalculate the life years. To account for time spent in suboptimal health due to IHD and any other conditions present, we calculated HALYs using estimates derived from prevalence and YLDs from IHD and from other causes. One HALY thus represents the equivalent of a year in perfect health. HALYs gained were calculated as the difference in HALYs between the reference and intervention populations. Changes in healthcare expenditures were estimated both for IHD-related healthcare and total healthcare. The change in IHD-related healthcare expenditure was based on the predicted reduction in IHD mortality and morbidity. Overall healthcare costs in added years of life were also included.^31^ Impact on health outcomes (ie, HALYs gained and averted or postponed IHD events and deaths) and healthcare cost savings were estimated over the total population and separately for women and men.

Net costs included policy costs and healthcare costs (including costs unrelated to IHD) and were used to calculate the incremental cost-effectiveness ratios (ICERs), defined as the difference in net costs of the intervention compared with current practice, divided by the difference in HALYs. We used WHO benchmarks for definition of cost-effectiveness, with a very cost-effective intervention being defined as ICER <US$1720 (ie, gross domestic product (GDP) per capita for Kenya in 2019) per HALY gained, and a cost-effective intervention being defined as ICER <US$5161 (ie, three times the GDP per capita) per HALY gained. Cost saving was defined as having a negative net cost.

#### Uncertainty and sensitivity analysis

The parameter uncertainty around the modelled estimates was quantified using Monte Carlo simulations (n=2000). For each iteration, a draw was made from the distributions of TFA intake, RRs, healthcare costs and policy implementation costs. The point estimate and 95% uncertainty intervals (UI) were defined as the 50th and 2.5th-97.5th percentiles, respectively, of the distribution of the intervention effects (eg, HALYs gained) estimated across all 2000 iterations using the Ersatz V.1.35 software. Similarly, Monte Carlo simulations (n=2000) of policy implementation costs were conducted in RStudio V.1.1.423.

Univariate sensitivity analysis was used to explore the impact of variation in discount rates (0% and 6%), TFA exposure and policy implementation costs (table 1). We evaluated the impact of higher postintervention intakes (0.10±0.01%E.) due to a higher intake of naturally occurring TFA or suboptimal compliance to the mandatory iTFA limit. We also evaluated the impact of 50% lower or higher mean and SD of preintervention intakes. It is possible that the prevalence of iTFA among foods in Kenya is greater than what was estimated (n=99 products) using the FoodSwitch database.^36^ In the sensitivity analysis, the number of products potentially containing iTFA was assumed to be twice as many (ie, n=198 products) as identified in the FoodSwitch database.^36^ Experience of TFA regulations in Denmark has suggested negligible reformulation costs^37^ and thus we conducted a sensitivity analysis assuming no industry costs. Given the differences between Tanzania (from where our assumption on government costs for the primary model was obtained) and Kenya, we also evaluated the impact of 50% greater monitoring costs compared with our primary analysis.

### Patient and public involvement

No patients were involved in setting the research question or the outcome measures, nor were they involved in developing plans for design or implementation of the study. No patients were asked to advise on interpretation or writing up of results.

## Results

### Main analysis

#### Health impact

**Figure 1:**
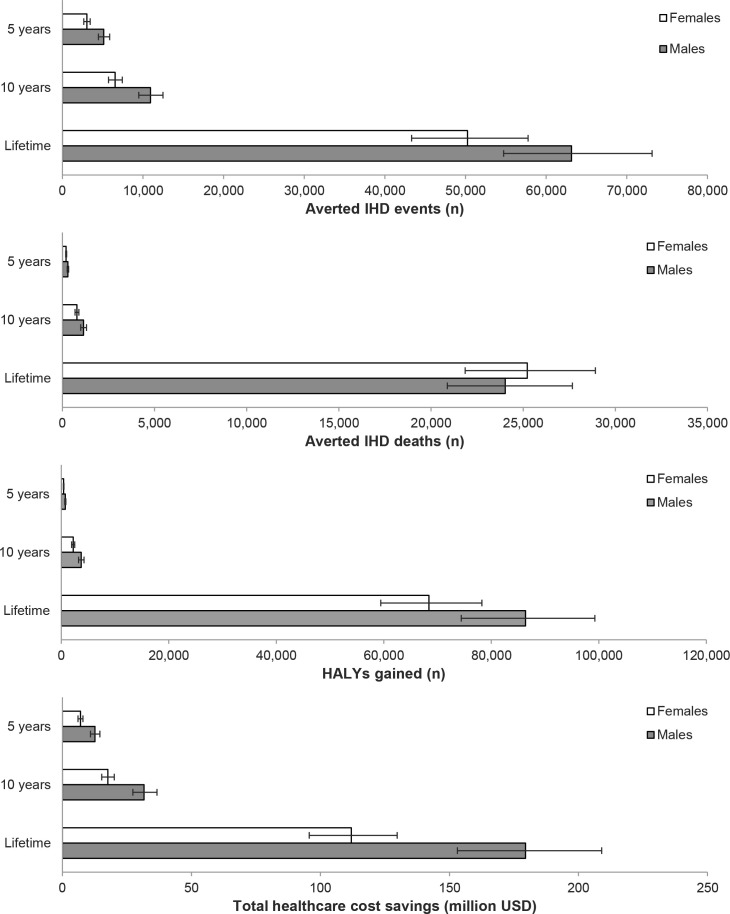
Sex-specific estimates of averted IHD events, averted IHD deaths, HALYs gained, and total healthcare cost savings after 5 years, 10 years, and over the population lifetime. Values are mean of n=2,000 simulations and error bars indicate 95% uncertainty intervals. HALYs, health-adjusted life years; IHD, ischaemic heart disease; USD, US dollar.

A mandatory limit of iTFA content in Kenyan foods was estimated to avert or postpone about 8200 incident IHD events and ~500 IHD deaths during the first 5 years, compared with a base case scenario maintaining current TFA intake levels (table 2). Over 10 years, around 17 000 incident events and ~1900 IHD deaths were estimated to be averted or postponed, and over the population lifetime (ie, the time from policy implementation until all individuals died or reached 100 years of age), around 110 000 IHD events and 49 000 IHD deaths could be averted (table 2). The iTFA limit was also estimated to generate about 1200 HALYs over the first 5 years, ~6000 HALYs in 10 years and ~150 000 HALYs over the population lifetime. In general, about 60–65% of the estimated health benefits accrued to men (figure 1). However, the IHD deaths averted over the population lifetime were more evenly distributed between women (51%) and men (49%).

**Table 2:** Estimated health and health economic effects of a mandatory limit of iTFA content (≤2% of all fats) in the Kenya food supply*

	Mean (95% UI)		
	5 years	10 years	Population lifetime
IHD incidence			
n	−8157 (−9116; −7248)	−17 454 (−19 551; −15 475)	−113 374 (−127 969; −100 104)
%†	−3.69 (−4.13; −3.26)	−3.60 (−4.03; −3.18)	−2.43 (−2.76; −2.13)
IHD deaths			
n	−499 (−556; −446)	−1926 (−2148; −1714)	−49 260 (−55 326; −43 775)
%†	−0.74 (−0.83; −0.66)	−1.30 (−1.45; −1.15)	−2.37 (−2.67; −2.09)
Health-adjusted life years, n	1175 (1044; 1310)	5891 (5228; 6568)	154 725 (136 607; 174 117)
IHD-related healthcare costs, million US$	−19.9 (−22.3; −17.6)	−51.3 (−57.8; −45.3)	−337 (−382; −296)
Total healthcare costs, million US$	−19.6 (−22.0; −17.3)	−49.3 (−55.6; −43.5)	−291 (−331; −256)
Total implementation costs, million US$	7.05 (5.24; 8.82)	9.12 (7.23; 10.99)	20.4 (18.1; 22.6)
Government implementation costs, million US$	2.45 (2.16; 2.74)	4.34 (3.97; 4.72)	14.6 (14.1; 15.2)
Industry reformulation costs, million US$	4.60 (2.81; 6.34)	4.78 (2.92; 6.59)	5.75 (3.51; 7.94)
Net costs, million US$	−12.5 (−15.5; −9.6)	−40.2 (−46.7; −34.1)	−271 (−310; −235)

*Outcomes are presented as mean and 95% UI defined as the 50th and 2.5th–97.5th percentiles, respectively. Negative values indicate reductions compared with the base case scenario, while positive values represent increases.

†Expressed as a percentage of IHD incident events or deaths under the base case scenario.

IHD, ischaemic heart disease; iTFA, industrial trans fatty acid; UI, uncertainty interval.

#### Economic impact

The total healthcare cost savings from the reduced iTFA intake and IHD burden were estimated as ~US$20 million (~Ksh2.0 billion) in the first 5 years, ~US$50 million (~Ksh5.1 billion) in 10 years and ~US$290 million (~Ksh30 billion) over the population lifetime (table 2). The lifetime healthcare cost savings specific to IHD were estimated as ~US$337 million (~Ksh35 billion).

Meanwhile, the implementation of the mandatory limit was estimated to cost the Kenyan government ~US$2.5 million (~Ksh250 million) in the first 5 years, accumulating to ~US$15 million (~Ksh1.5 billion) over the population lifetime (table 2 and online supplemental figure S2). The cost for industry to reformulate foods containing iTFA was estimated at around US$4.6 million (~Ksh470 million) in the first 5 years, US$4.8 million (~Ksh490 million) in 10 years and ~US$5.8 million (~Ksh590 million) over the population lifetime (table 2 and online supplemental figure S2). Thus, the total costs for the government and industry together were estimated as ~US$7.0 million (~Ksh720 million) in the first 5 years, ~US$9.1 million (~Ksh940 million) in 10 years and ~US$20 million (~Ksh2.1 billion) over the population lifetime (table 2).

As indicated above, the investment by the government in the implementation of the policy was estimated to generate a substantial return in healthcare cost savings. In the first 5 years, each US$ invested by the government could return about US$8 in healthcare savings, whereas over the population lifetime, the return of investment was estimated as US$20 saved per US$ invested.

#### Cost-effectiveness

The implementation of a mandatory limit of iTFA content in Kenyan foods was estimated to be net cost saving, with ~US$13 million saved in the first 5 years, ~US$40 million in 10 years and ~US$270 million saved over the population lifetime (table 2). When only industry and government policy implementation costs (but not healthcare cost savings) were considered, the mandatory limit of iTFA was already estimated to be cost-effective after 10 years (ICER: US$1553 (Ksh182 516) per HALY, 95% UI (US$1196; US$1937 (Ksh140 080; Ksh231 029) per HALY)), with 82% probability of being very cost-effective and 100% probability of being cost-effective. Over the population lifetime, the policy was estimated to be very cost-effective (ICER: US$132 (Ksh15 956) per HALY, 95% UI: US$111; US$155 (Ksh13 493; Ksh19 055) per HALY) even when healthcare cost savings were excluded, with 100% probability of being very cost-effective.

### Sensitivity analyses

**Figure 2:**
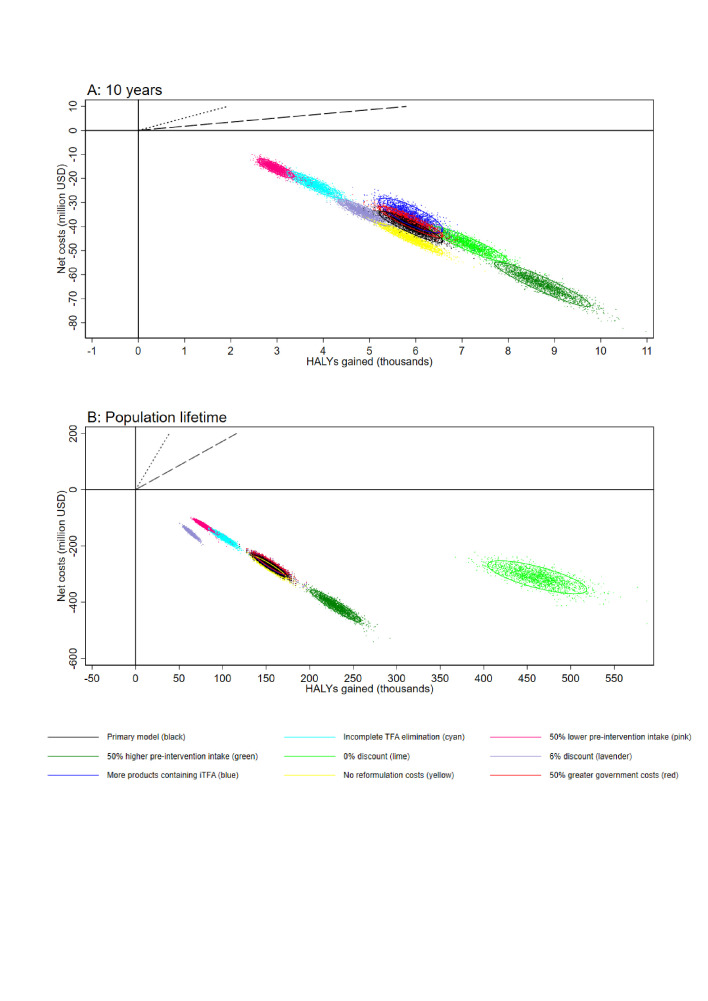
Net costs and HALYs gained during the first 10 years (A) and over the population lifetime (B) estimated in the primary model and in deterministic sensitivity analyses. Dotted and dashed black lines indicate thresholds for cost-effective and very cost-effective interventions, respectively. HALYs, health-adjusted life years; iTFA, industrial trans fatty acid; USD, US dollar.

The probability that the mandatory limit of iTFA content in Kenyan foods would be cost saving in the first 5 years was >99% for all sensitivity analyses and 100% over longer (≥10 years) time horizon (figure 2, online supplemental figure S3 and online supplemental table S9). Assumptions regarding preintervention and postintervention TFA intakes had the greatest impact on HALYs and net costs in the shorter time horizons (ie, ≤10 years), whereas assumptions on discount rates had the greatest impact on HALY and net cost estimates in the lifetime analyses. Assumptions regarding policy implementation costs (industry and government) had minimal impact on model estimates regardless of the time horizon modelled.

## Discussion

Using Markov cohort models with nationally representative data, we estimated the costs, impact on IHD burden and cost-effectiveness of a mandatory limit of iTFA (≤2% of total fat) in the Kenyan food supply compared with a base case scenario with no policy action to remove iTFA from foods to reduce TFA intake. The mandatory iTFA limit was estimated to prevent around 1900 deaths and 17 000 incident IHD events in the adult population (≥20 years) over the first 10 years, and around 49 000 deaths and 113 000 incident IHD events over the population’s lifetime. The intervention was estimated to be substantially cost saving over 5 years, 10 years as well as over the lifetime.

An iTFA limit in the Kenyan food supply could lead to considerable health benefits and a reduced IHD burden and healthcare costs. The estimated healthcare cost savings greatly outweighed the government’s costs for policy implementation (eightfold to 20-fold in the primary analysis), as well as any reformulation costs for the industry (in both primary and sensitivity analyses). Although not all savings would accrue to the public health system (given the significant utilisation of private healthcare in Kenya),^27^ the reduced spending on IHD care could potentially allow allocation of funds to non-IHD care or preventive public health campaigns, thereby further increasing the potential health gains from the modelled iTFA policy. Such additional funds could also be used to help eliminate any remaining iTFA in the informal food sector (eg, subsidise non-iTFA-containing oils and fats for street vendors). Although the prevalence of iTFA-containing ingredients in street foods in Kenya is unknown and heat-induced TFA accumulation during cooking of such foods is unlikely,^40^ there are some concerns that they could be a significant contributor of TFA intake in low and middle-income countries.^41^

The findings of a cost-saving strategy to prevent IHD mortality and morbidity were consistent over a series of sensitivity analyses with different inputs and assumptions regarding TFA intake, implementation costs and discount rate. Interestingly, our results showed that even when the policy did not lead to a complete elimination of TFA intake in Kenya, for example, if there is not a full compliance to the mandatory limit, the policy was still estimated to be a cost-saving strategy to generate substantive health gains.

Compared with previous cost-effectiveness analyses of policies to ban or limit iTFA in European countries and Australia, the estimated preintervention mean TFA intake in Kenyan adults was considerably lower (0.25–0.31%E vs ≥0.59%E). Still, the intervention was estimated to be overall cost saving. Even when healthcare savings were not considered, it could be very cost-effective within the first 10 years. Previous studies have suggested that mandatory iTFA limits (like the one modelled here) or bans of partially hydrogenated oils would outperform voluntary limits or mandatory labelling.^8^ Our findings expand this evidence by indicating that in Kenya, a lower middle-income African country with low estimated iTFA intake, a mandatory limit of iTFA in foods, oils and fats could even generate net cost savings. Such findings support national initiatives in Kenya, in other countries where iTFA intake is expected to be low, as well as the global call of WHO to eliminate iTFA from food supplies as a public health ‘best-buy’.^7^ Elimination of iTFA from the national food supply through legislation and other policy mechanisms is feasible. For example, iTFA was virtually eliminated in Denmark after legislation to limit the TFA content in foods.^9^ Mandatory TFA policies are in effect for at least 3.4 billion people in 60 countries (43% of the world population), with 43 of these countries having adopted WHO’s best practice policies, covering 2.8 billion people (36% of the world population).^42^ In recent years, many lower middle-income countries outside of Africa have adopted measures (India, Bangladesh, Philippines, Ukraine, Egypt). In addition, some market-leading global food companies and edible fat and oil suppliers have pledged to remove iTFA from their products,^43 44^ which suggests that even the food industry recognises that elimination of iTFA from foods is feasible. Still, similar voluntary actions by the food industry have often only partially reduced iTFA.^45^ Thus, government regulation to limit the use or to completely ban iTFA-containing ingredients, with monitoring of compliance and enforcement mechanisms, will likely be needed to ensure elimination of iTFA from the food supply.

This study has several strengths. We used a large country-specific database of packaged foods in Kenya to identify products potentially containing iTFA. The RR estimates of TFA intake with incident IHD were derived from a large meta-analysis of prospective studies directly linking consumption of TFA to incidence of IHD, thereby taking into consideration effects mediated by blood lipids and other potential pathways through which TFA intake can impact IHD, for example, inflammation. Our estimates of healthcare costs included both IHD-related and other healthcare costs, thereby allowing estimation of changes in total healthcare expenditures. We estimated health impact and healthcare cost savings separately for women and men.

Limitations of this study should also be considered. In the absence of nationally representative data on TFA intake in Kenya, we used estimates from the GBD study which used dietary data and partially hydrogenated vegetable oil sales data as inputs in a spatiotemporal Gaussian regression method to estimate TFA intakes by age, sex, country and year.^21^ Due to the scarceness of Kenya-specific cost data, we used costing frameworks from the UK (industry costs) and Tanzania (government costs), which may underestimate or overestimate such costs. Given the rapid increase in processed food consumption in sub-Saharan Africa,^46^ it is possible that iTFA intake, in the lack of an impactful policy, may also increase over the coming years and thus we may have underestimated the potential health gains while assuming a stable iTFA intake over the lifetime of the reference population (ie, base case scenario). Although experience from countries like Denmark suggests that a mandatory iTFA limit (≤2% of total fat) in foods, fats and oils will virtually eliminate iTFA intake, it may not be the case in Kenya. However, we evaluated a scenario of incomplete elimination in a sensitivity analysis, and the findings suggested that even if some TFA intake remained (mean: 0.10%E) after the intervention, the policy still had a 100% probability to be cost saving already after 5 years. The cost estimates used for industry reformulation were reported nearly 20 years ago, and it is possible that technological advances have allowed less costly reformulation processes given the increasing number of voluntary and mandatory measures globally to remove iTFA from the food supply. Furthermore, we did not include foods or ingredients not available in supermarkets in our estimation of potentially iTFA-containing products requiring reformulation. Still, in a sensitivity analysis assuming doubled number of reformulated products, the policy was estimated to be cost saving already after 5 years. Our estimation of healthcare cost savings did not include indirect costs (eg, productivity loss due to absenteeism or disability), and thus the societal savings from the intervention are likely to be substantially underestimated. We estimated effects stratified by sex, but due to scarceness of data (eg, on TFA intake or IHD burden), we were not able to further stratify our analyses on, for example, socioeconomic status or urban versus rural. Previous cost-effectiveness analyses have indicated that elimination of iTFA from the food supply could reduce socioeconomic and urban-rural inequalities in IHD disease burden in the UK and Australia.^12 34^ Given the differences between Kenya and high-income countries like the UK and Australia, it is unclear what impact such policy could have on inequalities in Kenya. Our model, in line with prior modelling papers, uses risk estimates of change in TFA against the overall diet rather than specific substitution with other fat classes. If iTFA were systematically replaced by saturated fatty acids, the impact of the mandatory limit could be lower than estimated here. However, evidence suggests no overall increase in saturated fatty acid content in food products as a result of iTFA elimination.^8 47^ This modelling study does not prove that a mandatory limit of iTFA will prevent IHD in Kenya; rather, it provides important quantitative estimates, corresponding uncertainty and assessments of sensitivity of the findings to different inputs, resulting in a range of plausible effects on IHD burden and cost-effectiveness of legislating a mandatory limit of iTFA in the Kenyan food supply to help inform policymakers.

## Conclusion

Compared with a base scenario with sustained intake of TFA at current levels, a mandatory best practice iTFA limit was estimated to be a cost-saving strategy to avert tens of thousands of IHD events and premature deaths in Kenya. Thus, our findings support initiatives to regulate iTFA content in the Kenyan food supply.

## Data Availability

All data relevant to the study are included in the article. Original data are available on reasonable request.
