# Supplementary material for: Estimated health benefits, costs and cost-effectiveness of eliminating dietary industrial trans fatty acids in Kenya: cost-effectiveness analysis
Source: BMJ Glob Health. 2023 Oct 17;8(10):e012692. doi: 10.1136/bmjgh-2023-012692 (PMC10583044; doi:10.1136/bmjgh-2023-012692)
Supplement: Supplementary data [file bmjgh-2023-012692supp001.pdf]

1. Supplementary methods

1.1. Study proposal/analytical plan

Study objective: To estimate the health impact, costs, and cost-effectiveness of mandatory limits of industrially-derived trans-fatty acid (iTFA) content in Kenyan foods.

Main policy scenarios: A limit of iTFA content (≤2% of all fats) in the Kenya food supply. The limit would target both packaged foods and foods purchased at restaurants or from street vendors.

Time horizon: (1) 10 years, and (2) lifetime

Model: Multiple cohort proportional multistate life table model

Population: Kenyan adults aged 20+ years

Perspective: Extended healthcare perspective (incorporating policy and direct healthcare costs)

Base-case scenario: No limits on iTFA in foods.

iTFA definition: Trans-fatty acids (TFA), not naturally occurring in foods, but produced during food processing.

Mandatory iTFA limit: ≤2% of all fats in foods.

Model outputs

- Health and economic outcomes: Ischemic heart disease (IHD) cases/deaths averted, health-adjusted life-years (HALY) gained, formal healthcare, policy costs (government costs for legislation and food monitoring; industry food reformulation costs), and cost-effectiveness (ICER).

Policy effects:

- We assume the changes in TFA intake to occur within one year after implementation.
- We assume there is no underlying ongoing (voluntary) industry reformulation to reduce iTFA content in foods available in the Kenya.
  - Based on mean intake estimates from the Global Burden of Disease study, no obvious trends in intake are observed in the period 2010-2019.
- In the main analysis, we assume that industry will comply fully with the mandatory limits immediately after policy implementation.
- In the main analysis, we assume that iTFA intake will be eliminated after the policy.[1]
- In the main analysis, we assume that intake of total TFA will be virtually eliminated with the removal of iTFA, given the minimal intake of naturally occurring TFA in Kenya compared to countries like Australia, UK, and Denmark.
- We will not account for any potential underlying trends in packaged or restaurant food intake.
- Reduction in TFA intake will reduce risk of IHD as estimated in previous meta-analysis within one year after policy implementation.[2]
- For the main analysis, we assume that the minimum risk level of trans-fat intake in Kenya is 0%E (i.e., equal to the post-policy intake).

Policy costs:

Regulatory/legislation costs for policy: We assume that policy legislation costs include parliamentarians' time, annual expenses for the National Assembly and the Senate, legislation drafting and publication, and policy advice.

Government cost for monitoring: We estimate monitoring costs using equivalent Kenyan shilling costs of UK estimates (annual [2011] cost of 2.4 million GBP), while adjusting for population size differences between the UK and Kenya.

Industry cost for reformulation and repackaging: We will calculate initial reformulation costs using equivalent Kenyan shilling costs from UK estimates (25,000 GBP per product) multiplied by the number of products in the Kenyan food supply potentially containing iTFA (primary model: n = X; sensitivity analysis: n = 2X).

- We assume that annual reformulation costs to be 1% of initial reformulation costs.
- We assume that the policy will result in increased costs to industry for changes in packaging and loss through disuse of existing packaging and we estimate this as a 1-time cost equivalent to 10% of the initial reformulation costs

Sensitivity & Stratified Analysis:

- **Stratified analyses:**
  - Sex
- **Deterministic sensitivity analysis**
  - Higher or lower pre-policy intake
  - Higher post policy intake
  - Alternative distributions of transfat intake
  - Alternative theoretical minimum risk level/distribution
  - Higher or lower discount rate
  - Higher reformulation costs
- **Probability sensitivity analysis** will be conducted for the main analysis and each of the deterministic sensitivity analyses, incorporating uncertainty for relevant data inputs.

1.2. DisMod II

The proportional multistate lifetable (pMSLT) model required input parameters: incidence, prevalence, remission and case fatality rates for each disease. Case fatality rates are not provided by the GBD study and were derived using DISMOD II software.[3] Given the chronic nature of Ischemic heart disease (IHD), remission was set to zero. In the DisMod II process, various adjustments were applied based on options given in the software. For both males and females, we set remission input as exact zero. Further, in males, the prevalence weighting was increased to the midpoint between low and medium point.

The conceptual model of DisMod II is that of a multi-state lifetable model where "*Healthy people, defined as people unaffected by the disease being modelled, are subject to an incidence hazard, and may become diseased. When diseased they are subject to a hazard of dying from the disease, the case fatality, and to a hazard of recovery from the disease, called remission. Both healthy and diseased people are subject to the same mortality hazard from all other causes*" (eFigure 1).[3]

DisMod II generates outputs (incidence, prevalence, remission, case fatality) presented by sex and one-year age groups (Table A3). The outputs are in rates per one and are used in the pMSLT model as disease inputs.

1.3. Calculation of disability weights

Disability weights (DW) indicate the average loss of quality of life due to disease [4]. We calculated DW by dividing the number of IHD-specific years lived with disability (YLDs) by the number of prevalent cases of IHD for each age/sex group. We then adjusted the DWs for disability due to other diseases in each age group.

We did this by inflating the result to account for the fact that due to the presence of other health conditions, the starting point for the calculation of the YLDs was less than full health. We inflated by '1-pYLD / pop', whereby pYLD stands for the total YLD for all diseases except the one under scrutiny (IHD), and pop is the population number. This calculation is done to derive DWs at the cause level (e.g., IHD) instead of sequela level (e.g., myocardial infarction [MI], chronic IHD, angina, asymptomatic IHD following MI, acute MI) as reported in the Global Burden of Disease (GBD) study. We base our calculations on the GBD methods for estimating YLDs as the disease prevalence multiplied by disability weights. The DWs enable quantification of health levels associated with non-fatal outcomes and are used in the calculation of health-adjusted life years [4]. eTables 5-A show the DW and pYLD used in the model.

2. Supplementary references

1. Downs, S.M., et al., *The Impact of Policies to Reduce trans Fat Consumption: A Systematic Review of the Evidence.* Current Developments in Nutrition, 2017. **1**(12).
2. Wang, Q., et al., *Impact of Nonoptimal Intakes of Saturated, Polyunsaturated, and Trans Fat on Global Burdens of Coronary Heart Disease.* 2016. **5**(1): p. e002891.
3. Barendregt, J.J., et al., *A generic model for the assessment of disease epidemiology: the computational basis of DisMod II.* Population Health Metrics, 2003. **1**(1): p. 4.
4. Salomon, J.A., et al., *Disability weights for the Global Burden of Disease 2013 study.* The Lancet Global Health, 2015. **3**(11): p. e712-e723.
5. Global Burden of Disease Collaborative Network, *Global Burden of Disease Study 2019 (GBD 2019) Results*. 2020, Seattle, United States of America: Institute for Health Metrics and Evaluation (IHME).

Table S1. Baseline (pre-policy) in intake of total TFA by age and sex.*

		Pre-policy intake (%E)†	
**Sex**	**Age (y)**	**Mean**	**SD**
Women	20-24	n/a‡	n/a[1]
	25-29	0.25 (0.17; 0.35)	0.14 (0.09; 0.19)
	30-34	0.25 (0.17; 0.35)	0.14 (0.09; 0.20)
	35-39	0.25 (0.18; 0.36)	0.14 (0.09; 0.20)
	40-44	0.26 (0.18; 0.37)	0.14 (0.10; 0.20)
	45-49	0.27 (0.19; 0.38)	0.14 (0.10; 0.21)
	50-54	0.27 (0.18; 0.37)	0.15 (0.10; 0.21)
	55-59	0.27 (0.19; 0.37)	0.15 (0.10; 0.20)
	60-64	0.28 (0.20; 0.38)	0.15 (0.10; 0.21)
	65-69	0.28 (0.19; 0.40)	0.15 (0.10; 0.22)
	70-74	0.29 (0.20; 0.42)	0.16 (0.11; 0.23)
	75-79	0.30 (0.20; 0.41)	0.16 (0.11; 0.23)
	80-84	0.31 (0.21; 0.43)	0.17 (0.11; 0.24)
	85-89	0.30 (0.21; 0.43)	0.17 (0.11; 0.24)
	90-94	0.30 (0.21; 0.42)	0.17 (0.11; 0.23)
	95+	0.31 (0.21; 0.44)	0.17 (0.11; 0.24)
Men	20-24	n/a[1]	n/a[1]
	25-29	0.25 (0.18; 0.35)	0.14 (0.09; 0.19)
	30-34	0.25 (0.17; 0.35)	0.14 (0.09; 0.20)
	35-39	0.26 (0.18; 0.36)	0.14 (0.10; 0.20)
	40-44	0.26 (0.18; 0.36)	0.14 (0.10; 0.20)
	45-49	0.26 (0.18; 0.36)	0.14 (0.10; 0.20)
	50-54	0.27 (0.18; 0.37)	0.15 (0.10; 0.20)
	55-59	0.27 (0.19; 0.39)	0.15 (0.10; 0.21)
	60-64	0.28 (0.19; 0.39)	0.15 (0.10; 0.22)
	65-69	0.28 (0.20; 0.39)	0.15 (0.10; 0.22)
	70-74	0.29 (0.20; 0.41)	0.16 (0.11; 0.22)
	75-79	0.29 (0.20; 0.41)	0.16 (0.11; 0.23)
	80-84	0.31 (0.21; 0.42)	0.17 (0.11; 0.24)
	85-89	0.31 (0.22; 0.43)	0.17 (0.12; 0.24)
	90-94	0.30 (0.21; 0.42)	0.17 (0.11; 0.23)
	95+	0.31 (0.21; 0.44)	0.17 (0.11; 0.24)

*%E, energy percentage; SD, standard deviation. †Estimates from Afshin et al. Lancet, 2019; 393 (10184):1958 - 1972. ‡Estimates for ages 20-24 years not available, thus estimates for ages 25-29 years used for both age groups.

Table S2. Population size and mortality rate, by sex and age.

	Population size (1000s)		Mortality rate (per 1000)	
**Age**	**Women**	**Men**	**Women**	**Men**
20	534.6	533.1	1.3	2.1
21	517.5	510.8	1.4	2.1
22	501.0	489.6	1.4	2.1
23	484.9	469.6	1.5	2.1
24	469.1	450.6	1.7	2.1
25	453.8	432.5	1.8	2.2
26	439.7	416.4	2.0	2.2
27	426.8	402.1	2.2	2.4
28	415.1	389.7	2.4	2.5
29	404.6	379.1	2.6	2.7
30	395.1	370.3	2.8	2.8
31	385.1	361.3	3.0	3.1
32	374.6	352.1	3.2	3.3
33	363.5	342.4	3.5	3.6
34	351.2	332.3	3.7	3.9
35	337.5	321.8	4.0	4.3
36	324.3	311.0	4.3	4.7
37	311.3	300.1	4.6	5.2
38	298.3	289.1	4.9	5.6
39	285.1	277.9	5.2	6.1
40	271.6	266.6	5.5	6.6
41	258.1	255.3	5.9	7.1
42	245.2	244.2	6.2	7.7
43	233.1	233.6	6.6	8.3
44	222.0	223.2	6.9	8.9
45	212.0	213.2	7.3	9.5
46	201.6	203.3	7.7	10.1
47	191.2	193.7	8.1	10.8
48	181.0	184.2	8.5	11.5
49	171.3	174.9	8.8	12.3
50	162.4	165.9	9.2	13.1
51	154.0	157.3	9.6	13.9
52	146.3	149.2	10.0	14.8
53	139.1	141.5	10.3	15.7
54	132.4	134.3	10.7	16.6
55	126.1	127.5	11.1	17.6
56	120.5	121.2	11.6	18.7
57	115.3	115.3	12.2	19.9
58	110.3	109.5	12.9	21.3
59	105.3	104.0	13.7	22.8
60	100.2	98.5	14.6	24.5
61	94.6	92.4	15.6	26.3
62	88.5	85.7	16.8	28.2
63	82.8	79.6	18.0	30.1
64	77.6	74.0	19.3	32.2
65	72.8	69.2	20.8	34.4
66	68.8	65.0	22.5	36.8
67	65.3	61.5	24.5	39.5
68	61.9	57.8	26.8	42.5
69	51.3	50.1	29.4	46.0
70	51.0	47.6	32.4	49.8
71	48.8	43.6	35.8	54.0
72	44.9	38.4	39.5	58.5
73	41.5	34.7	43.5	63.2
74	38.1	31.6	47.7	68.1
75	34.3	28.9	52.4	73.5

76	31.4	25.4	57.6	79.3
77	29.2	21.4	63.4	85.8
78	26.4	18.2	69.9	93.0
79	23.6	15.5	77.2	101.0
80	20.5	13.5	85.3	109.7
81	18.2	11.5	93.8	119.1
82	16.5	9.7	102.7	129.0
83	14.5	8.2	111.7	139.3
84	12.6	6.8	120.9	150.1
85	10.5	5.6	130.7	161.8
86	8.8	4.5	141.4	174.8
87	7.4	3.6	153.4	189.6
88	6.1	2.8	167.3	206.6
89	4.9	2.2	182.0	224.6
90	4.0	1.6	196.0	241.5
91	3.2	1.2	207.0	254.9
92	2.4	0.9	212.7	261.7
93	1.9	0.7	210.8	259.4
94	1.4	0.5	199.8	246.0
95	1.0	0.3	243.0	282.4
96	0.8	0.2	254.7	292.0
97	0.6	0.2	266.2	301.1
98	0.5	0.1	277.4	309.5
99	0.4	0.1	288.4	317.3
100+	0.3	0.1	-*	-*

*Model simulations terminated when all individuals were dead or had reached the age 100 years.

Table S3. Estimated rates of IHD incidence and prevalence (per 100K) and IHD case fatality by age and sex.

	Women			Men		
Age	Incidence (per 100K)	Prevalence (per 100K)	Case fatality (%)	Incidence (per 100K)	Prevalence (per 100K)	Case fatality (%)
20	3.62	17.71	4.4%	6.09	25.57	5.6%
21	4.44	20.91	4.1%	7.84	31.01	5.2%
22	5.49	24.97	3.8%	10.09	38.23	4.9%
23	6.78	30.13	3.4%	12.84	47.71	4.4%
24	8.31	36.64	2.9%	16.08	59.95	3.9%
25	10.15	44.80	2.4%	19.89	75.49	3.4%
26	12.07	54.80	2.0%	23.77	94.64	2.9%
27	14.06	66.72	1.7%	27.71	117.46	2.6%
28	16.12	80.62	1.5%	31.73	143.96	2.3%
29	18.26	96.57	1.3%	35.81	174.17	2.1%
30	20.47	114.59	1.2%	39.96	208.06	2.0%
31	22.81	134.70	1.2%	44.34	245.60	2.0%
32	25.21	156.93	1.2%	48.89	286.87	2.0%
33	27.66	181.31	1.2%	53.60	331.97	1.9%
34	30.16	207.82	1.2%	58.48	380.98	1.9%
35	32.71	236.49	1.2%	63.53	433.99	1.9%
36	35.32	267.30	1.3%	68.74	491.09	1.9%
37	38.61	300.55	1.3%	75.43	552.99	1.9%
38	42.54	336.78	1.3%	83.44	620.95	1.9%
39	47.10	376.55	1.4%	92.76	696.13	1.9%
40	52.29	420.34	1.5%	103.40	779.64	1.9%
41	58.12	468.66	1.5%	115.35	872.57	1.9%
42	64.57	521.96	1.6%	128.62	975.97	1.9%
43	72.50	581.10	1.7%	144.75	1,091.60	1.9%
44	81.27	647.04	1.7%	162.44	1,221.25	1.9%
45	90.88	720.45	1.8%	181.68	1,366.02	2.0%
46	101.33	801.93	1.9%	202.46	1,526.95	2.0%
47	112.61	892.11	1.9%	224.80	1,705.00	2.0%
48	124.74	991.59	2.0%	248.69	1,901.09	2.1%
49	138.91	1,101.57	2.0%	276.68	2,117.29	2.1%
50	154.31	1,223.41	2.0%	307.22	2,356.10	2.1%
51	170.91	1,357.98	2.0%	340.31	2,619.22	2.2%
52	188.73	1,506.16	2.1%	375.95	2,908.22	2.2%
53	207.77	1,668.78	2.1%	414.14	3,224.63	2.3%
54	228.02	1,846.67	2.1%	454.88	3,569.85	2.3%
55	251.56	2,041.57	2.1%	501.87	3,946.85	2.3%
56	277.19	2,255.46	2.1%	552.56	4,358.82	2.4%
57	304.89	2,489.59	2.2%	606.95	4,807.47	2.4%
58	334.66	2,745.08	2.2%	665.04	5,294.25	2.5%
59	366.52	3,022.94	2.3%	726.83	5,820.39	2.5%
60	400.45	3,324.04	2.3%	792.33	6,386.84	2.6%
61	437.19	3,649.32	2.4%	861.95	6,994.77	2.7%
62	474.66	3,998.84	2.5%	932.01	7,644.06	2.7%
63	512.86	4,371.63	2.5%	1,002.51	8,333.09	2.8%
64	551.78	4,766.59	2.6%	1,073.43	9,060.32	2.8%
65	591.43	5,182.50	2.7%	1,144.79	9,824.35	2.8%
66	631.81	5,618.00	2.9%	1,216.58	10,623.88	2.8%
67	675.34	6,072.34	3.0%	1,290.92	11,458.31	2.9%
68	721.30	6,545.18	3.1%	1,367.37	12,327.01	2.9%
69	769.68	7,035.80	3.3%	1,445.93	13,228.57	2.9%
70	820.49	7,543.24	3.5%	1,526.61	14,161.43	3.0%
71	873.72	8,066.31	3.7%	1,609.40	15,123.81	3.0%
72	929.37	8,603.58	3.9%	1,694.30	16,113.77	3.1%
73	988.79	9,153.30	4.1%	1,781.08	17,128.75	3.2%
74	1,049.54	9,712.77	4.4%	1,867.62	18,165.21	3.2%

Marklund M, *et al. BMJ Glob Health* 2024; 8:e012692. doi: 10.1136/bmjgh-2023-012692

75	1,111.63	10,278.57	4.7%	1,953.92	19,219.24	3.3%
76	1,175.05	10,847.17	4.9%	2,039.98	20,286.99	3.4%
77	1,239.81	11,414.99	5.3%	2,125.80	21,364.70	3.5%
78	1,305.91	11,978.43	5.6%	2,211.38	22,448.71	3.6%
79	1,372.05	12,530.38	6.0%	2,288.25	23,529.18	3.8%
80	1,436.90	13,060.63	6.4%	2,356.64	24,590.83	3.9%
81	1,500.46	13,559.50	7.0%	2,416.55	25,619.40	4.1%
82	1,562.73	14,017.69	7.5%	2,467.98	26,601.15	4.4%
83	1,623.71	14,426.49	8.2%	2,510.94	27,522.91	4.7%
84	1,683.39	14,777.97	8.8%	2,545.41	28,372.21	5.0%
85	1,740.21	15,060.97	9.6%	2,567.86	29,127.81	5.4%
86	1,795.44	15,265.48	10.5%	2,586.75	29,766.48	5.9%
87	1,849.09	15,387.22	11.4%	2,602.09	30,272.52	6.5%
88	1,901.15	15,423.63	12.4%	2,613.89	30,631.30	7.2%
89	1,951.64	15,373.95	13.4%	2,622.12	30,829.56	8.0%
90	2,000.54	15,239.35	14.5%	2,626.81	30,855.70	8.8%
91	2,046.35	15,021.56	15.7%	2,639.65	30,708.55	9.7%
92	2,090.66	14,727.04	16.8%	2,664.20	30,406.68	10.5%
93	2,133.47	14,367.77	18.0%	2,700.45	29,978.98	11.3%
94	2,174.77	13,956.08	19.2%	2,748.40	29,453.99	12.1%
95	2,214.56	13,504.29	20.4%	2,808.06	28,859.56	12.8%
96	2,252.85	13,024.25	21.7%	2,879.42	28,222.50	13.4%
97	2,284.38	12,533.68	22.7%	2,950.62	27,572.29	14.0%
98	2,319.07	12,045.00	23.9%	3,028.94	26,925.67	14.5%
99	2,350.95	11,565.83	25.0%	3,100.93	26,287.00	15.1%
100+	2,363.21	11,127.24	25.4%	3,128.62	25,676.49	15.3%

Marklund M, *et al. BMJ Glob Health* 2024; 8:e012692. doi: 10.1136/bmjgh-2023-012692

Table S4. Etiologic effects of Relative risk for IHD per 2%E from TFA.[*†]

Age (y)	Relative risk (95% confidence interval)
25-29	1.901 (1.591-2.275)
30-34	1.775 (1.514-2.085)
35-39	1.615 (1.415-1.848)
40-44	1.517 (1.352-1.707)
45-49	1.461 (1.316-1.627)
50-54	1.396 (1.274-1.535)
55-59	1.323 (1.225-1.433)
60-64	1.264 (1.186-1.352)
65-69	1.222 (1.157-1.294)
70-74	1.186 (1.132-1.246)
75-79	1.158 (1.112-1.207)
80-84	1.150 (1.107-1.197)
85-89	1.150 (1.107-1.197)
90-94	1.150 (1.107-1.197)
95+	1.150 (1.107-1.197)

[*]%E, energy percentage; TFA, trans-fatty acids; IHD, ischemic heart disease. †Estimates from Afshin et al. Lancet, 2019; 393 (10184):1958 - 1972.

Table S5. Age- and sex-specific disability weights for IHD adjusted for disability from other causes.

Age	Male	Female
Under 5	0.00	0.00
5 to 9	0.00	0.00
10 to 14	0.00	0.00
15 to 19	0.02	0.02
20 to 24	0.03	0.03
25 to 29	0.03	0.03
30 to 34	0.03	0.03
35 to 39	0.04	0.03
40 to 44	0.04	0.04
45 to 49	0.05	0.04
50 to 54	0.05	0.04
55 to 59	0.04	0.04
60 to 64	0.05	0.04
65 to 69	0.05	0.04
70 to 74	0.05	0.05
75 to 79	0.05	0.05
80 to 84	0.06	0.05
85 to 89	0.06	0.05
90 to 94	0.07	0.06
95 plus	0.07	0.08

Table S6. All-cause prevalent years lived with disability (pYLD) rates by sex and single-year age groups

Age	Male	Female	Age	Male	Female	Age	Male	Female	Age	Male	Female
**0**	0.04	0.04	**26**	0.08	0.10	**51**	0.14	0.17	**76**	0.27	0.26
**1**	0.04	0.04	**27**	0.08	0.11	**52**	0.15	0.17	**77**	0.27	0.27
**2**	0.04	0.04	**28**	0.08	0.11	**53**	0.15	0.17	**78**	0.28	0.27
**3**	0.04	0.04	**29**	0.09	0.12	**54**	0.15	0.17	**79**	0.29	0.28
**4**	0.04	0.04	**30**	0.09	0.12	**55**	0.16	0.17	**80**	0.29	0.28
**5**	0.04	0.04	**31**	0.09	0.12	**56**	0.16	0.18	**81**	0.30	0.29
**6**	0.04	0.04	**32**	0.09	0.13	**57**	0.17	0.18	**82**	0.31	0.30
**7**	0.04	0.04	**33**	0.09	0.13	**58**	0.17	0.18	**83**	0.31	0.30
**8**	0.05	0.04	**34**	0.10	0.13	**59**	0.17	0.18	**84**	0.32	0.31
**9**	0.05	0.04	**35**	0.10	0.13	**60**	0.18	0.19	**85**	0.32	0.31
**10**	0.05	0.05	**36**	0.10	0.14	**61**	0.18	0.19	**86**	0.33	0.32
**11**	0.05	0.05	**37**	0.10	0.14	**62**	0.18	0.19	**87**	0.33	0.32
**12**	0.05	0.05	**38**	0.11	0.14	**63**	0.19	0.20	**88**	0.34	0.33
**13**	0.05	0.06	**39**	0.11	0.14	**64**	0.19	0.20	**89**	0.34	0.33
**14**	0.06	0.06	**40**	0.11	0.15	**65**	0.20	0.20	**90**	0.35	0.34
**15**	0.06	0.06	**41**	0.11	0.15	**66**	0.20	0.21	**91**	0.35	0.34
**16**	0.06	0.07	**42**	0.12	0.15	**67**	0.21	0.21	**92**	0.36	0.35
**17**	0.06	0.07	**43**	0.12	0.15	**68**	0.22	0.22	**93**	0.37	0.36
**18**	0.06	0.07	**44**	0.12	0.16	**69**	0.22	0.22	**94**	0.37	0.37
**19**	0.06	0.08	**45**	0.12	0.16	**70**	0.23	0.23	**95**	0.38	0.37
**20**	0.07	0.08	**46**	0.13	0.16	**71**	0.24	0.23	**96**	0.39	0.38
**21**	0.07	0.08	**47**	0.13	0.16	**72**	0.24	0.24	**97**	0.39	0.38
**22**	0.07	0.09	**48**	0.13	0.16	**73**	0.25	0.25	**98**	0.39	0.38
**23**	0.07	0.09	**49**	0.14	0.16	**74**	0.26	0.25	**99**	0.39	0.38
**24**	0.07	0.10	**50**	0.14	0.16	**75**	0.26	0.26	**100**	0.39	0.38
**25**	0.08	0.10									

Years lived with disability (YLDs) and Kenya population data estimates from the 2019 GBD study used to calculate per person YLD [5].

Table S7. Total, IHD-related, and other healthcare costs per capita, by sex and age.

	Annual pre-intervention healthcare costs per capita, USD						
	Women			Males			
Age	Total	IHD	Non-IHD	Total	IHD	Non-IHD
20	31.4	0.1	31.3	22.3	0.2	22.1
21	31.4	0.1	31.3	22.3	0.3	22.1
22	31.4	0.2	31.3	22.4	0.3	22.1
23	31.5	0.2	31.3	22.5	0.4	22.1
24	31.5	0.2	31.3	22.6	0.5	22.1
25	45.2	0.3	44.9	33.7	0.7	33.0
26	45.3	0.4	44.9	33.8	0.8	33.0
27	45.4	0.4	44.9	34.0	1.0	33.0
28	45.4	0.5	44.9	34.2	1.2	33.0
29	45.5	0.6	44.9	34.4	1.4	33.0
30	45.6	0.7	44.9	34.6	1.6	33.0
31	45.7	0.8	44.9	34.8	1.8	33.0
32	45.9	1.0	44.9	35.1	2.1	33.0
33	46.0	1.1	44.9	35.4	2.4	33.0
34	46.2	1.2	44.9	35.7	2.7	33.0
35	85.4	1.4	84.0	61.0	3.0	58.0
36	85.6	1.6	84.0	61.3	3.4	58.0
37	85.7	1.7	84.0	61.7	3.7	58.0
38	85.9	2.0	84.0	62.2	4.2	58.0
39	86.2	2.2	84.0	62.7	4.7	58.0
40	86.4	2.4	84.0	63.2	5.2	58.0
41	86.7	2.7	84.0	63.8	5.9	58.0
42	87.0	3.0	84.0	64.5	6.5	58.0
43	87.3	3.4	84.0	65.3	7.3	58.0
44	87.7	3.7	84.0	66.2	8.2	58.0
45	214.6	4.2	210.4	146.3	9.2	137.2
46	215.0	4.7	210.4	147.4	10.3	137.2
47	215.6	5.2	210.4	148.6	11.4	137.2
48	216.1	5.8	210.4	149.9	12.7	137.2
49	216.8	6.4	210.4	151.3	14.2	137.2
50	217.5	7.1	210.4	152.9	15.7	137.2
51	218.3	7.9	210.4	154.6	17.5	137.2
52	219.2	8.8	210.4	156.5	19.4	137.2
53	220.1	9.7	210.4	158.6	21.4	137.2
54	221.1	10.7	210.4	160.8	23.7	137.2
55	548.0	11.8	536.2	386.5	26.2	360.4
56	549.3	13.1	536.2	389.2	28.9	360.4
57	550.6	14.4	536.2	392.1	31.8	360.4
58	552.1	15.9	536.2	395.3	35.0	360.4
59	553.7	17.5	536.2	398.7	38.4	360.4
60	555.5	19.3	536.2	402.4	42.0	360.4
61	557.3	21.2	536.2	406.3	45.9	360.4
62	559.4	23.2	536.2	410.4	50.0	360.4
63	561.5	25.4	536.2	414.7	54.3	360.4
64	563.8	27.6	536.2	419.1	58.8	360.4
65	958.8	30.0	928.8	808.6	63.4	745.2
66	961.3	32.5	928.8	813.4	68.2	745.2
67	963.9	35.1	928.8	818.4	73.3	745.2
68	966.6	37.8	928.8	823.6	78.5	745.2
69	969.4	40.7	928.8	829.0	83.9	745.2
70	972.4	43.7	928.8	834.6	89.4	745.2
71	975.5	46.7	928.8	840.3	95.2	745.2
72	978.7	49.9	928.8	846.2	101.1	745.2

73	982.0	53.2	928.8	852.3	107.1	745.2
74	985.4	56.6	928.8	858.4	113.2	745.2
75	988.8	60.1	928.8	864.6	119.5	745.2
76	992.3	63.6	928.8	870.9	125.7	745.2
77	995.9	67.1	928.8	877.2	132.0	745.2
78	999.4	70.7	928.8	883.6	138.4	745.2
79	1,003.0	74.3	928.8	889.7	144.6	745.2
80	1,006.5	77.8	928.8	895.7	150.5	745.2
81	1,009.9	81.2	928.8	901.4	156.2	745.2
82	1,013.1	84.4	928.8	906.7	161.5	745.2
83	1,016.2	87.4	928.8	911.6	166.4	745.2
84	1,018.9	90.2	928.8	916.0	170.9	745.2
85	1,021.4	92.7	928.8	919.8	174.7	745.2
86	1,023.6	94.8	928.8	923.1	177.9	745.2
87	1,025.3	96.6	928.8	925.6	180.5	745.2
88	1,026.7	98.0	928.8	927.5	182.3	745.2
89	1,027.6	98.9	928.8	928.5	183.3	745.2
90	1,028.2	99.4	928.8	928.7	183.5	745.2
91	1,028.3	99.5	928.8	928.2	183.1	745.2
92	1,028.0	99.2	928.8	927.2	182.1	745.2
93	1,027.3	98.5	928.8	925.9	180.7	745.2
94	1,026.3	97.5	928.8	924.3	179.1	745.2
95	1,025.0	96.3	928.8	922.5	177.3	745.2
96	1,023.6	94.8	928.8	920.8	175.6	745.2
97	1,021.9	93.2	928.8	918.9	173.8	745.2
98	1,020.2	91.4	928.8	917.2	172.1	745.2
99	1,018.5	89.7	928.8	915.5	170.3	745.2
100+	1,016.7	88.0	928.8	913.2	168.0	745.2

Marklund M, *et al. BMJ Glob Health* 2024; 8:e012692. doi: 10.1136/bmjgh-2023-012692

Table S8. Undiscounted policy costs in US dollar for government and industry by year.

	Model	Year 1	Year 2	Year 3	Year 4	Year 5	Year 6+
**Government costs (US dollars)[1]**							
Strategy development and evaluation (including development and legislation of laws)	Primary model	52,710	0	0	0	0	0
	Sensitivity analysis	79,065	0	0	0	0	0
Human resources (for program management and law enforcement)	Primary model	305,199	301,151	305,199	301,091	301,091	301,091
	Sensitivity analysis	457,799	451,727	457,799	451,637	451,637	451,637
Promotion and media advocacy	Primary model	69,837	0	37,430	0	0	0
	Sensitivity analysis	104,756	0	56,145	0	0	0
Office rent, equipment and supplies	Primary model	6,278	5,409	6,278	5,409	5,409	0
	Sensitivity analysis	9,417	8,114	9,417	8,114	8,114	0
Administration	Primary model	197,967	165,852	188,428	166,943	165,852	165,852
	Sensitivity analysis	296,951	248,778	282,642	250,415	248,778	248,778
**Industry costs (US dollars)[2]**							
Initial reformulation	Primary model	4,437,750	0	0	0	0	0
	Sensitivity analysis	8,875,500	0	0	0	0	0
Ongoing reformulation	Primary model	0	44,378	44,378	44,378	44,378	44,378
	Sensitivity analysis	0	88,755	88,755	88,755	88,755	88,755

[1]Government costs were based on estimates from a robust costing study conducted as part of a cost-effectiveness analysis of tobacco control policies in Tanzania (Ngalesoni et al. Cost-effectiveness analysis of population-based tobacco control strategies in the prevention of cardiovascular diseases in Tanzania. PLoS One 2017;12(8):e0182113.) [2]Industry reformulation costs were estimated using equivalent USD costs from UK estimates (£25,000 per product) multiplied by the number of products in the Kenyan market potentially containing iTFA (Allen et al. Potential of trans fats policies to reduce socioeconomic inequalities in mortality from coronary heart disease in England: cost effectiveness modelling study. BMJ 2015;351:h4583; and Huang et al. Presence of trans-Fatty Acids Containing Ingredients in Pre-Packaged Foods and the Availability of Reported trans-Fat Levels in Kenya and Nigeria. *Nutrients* 2023;15(3):761).

Table S9. Estimated cost-effectiveness of implementing a mandatory limit of iTFA content (≤2% of all fats) in the Kenya food supply, in main analysis and deterministic sensitivity analyses.

	Main analysis	Incomplete elimination	Lower pre-intervention intake	Higher pre-intervention intake	0% discount	6% discount	More products reformulated	No reformulation costs	50% greater government costs
5 years									
HALYs gained, thousands	1.17 (1.05; 1.31)	0.76 (0.65; 0.88)	0.59 (0.52; 0.68)	1.74 (1.56; 1.95)	1.28 (1.14; 1.43)	1.08 (0.96; 1.21)	1.17 (1.05; 1.31)	1.17 (1.05; 1.31)	1.17 (1.05; 1.31)
Net costs, million USD	-12.5 (-15.5; -9.6)	-5.6 (-8.5; -3.0)	-2.8 (-5.2; -0.7)	-22.0 (-26.2; -18.3)	-13.7 (-17.0; -10.7)	-11.4 (-14.3; -8.7)	-7.9 (-12.2; -3.6)	-17.1 (-19.5; -14.8)	-11.3 (-14.3; -8.4)
ICER, USD per HALY gained[2]	dominant (dominant; dominant)	dominant (dominant; dominant)	dominant (dominant; dominant)	dominant (dominant; dominant)	dominant (dominant; dominant)	dominant (dominant; dominant)	dominant (dominant; dominant)	dominant (dominant; dominant)	dominant (dominant; dominant)
Probabilities									
Cost saving	100%	100%	99.6%	100%	100%	100%	100%	100%	100%
Very cost-effective	100%	100%	100%	100%	100%	100%	100%	100%	100%
Cost-effective	100%	100%	100%	100%	100%	100%	100%	100%	100%
10 years									
HALYs gained, thousands	5.89 (5.23; 6.57)	3.80 (3.27; 4.40)	2.97 (2.63; 3.39)	8.74 (7.79; 9.82)	7.13 (6.33; 7.98)	4.89 (4.36; 5.51)	5.89 (5.23; 6.57)	5.89 (5.23; 6.57)	5.89 (5.23; 6.57)
Net costs, million USD	-40.2 (-46.7; -34.1)	-22.6 (-28.6; -17.5)	-15.8 (-20.1; -12.3)	-64.0 (-73.5; -55.5)	-47.2 (-54.8; -40.2)	-34.2 (-39.7; -29.2)	-35.4 (-42.4; -28.5)	-44.9 (-51.1; -39.1)	-38.0 (-44.4; -31.9)
ICER, USD per HALY gained[2]	dominant (dominant; dominant)	dominant (dominant; dominant)	dominant (dominant; dominant)	dominant (dominant; dominant)	dominant (dominant; dominant)	dominant (dominant; dominant)	dominant (dominant; dominant)	dominant (dominant; dominant)	dominant (dominant; dominant)
Probabilities									
Cost saving	100%	100%	100%	100%	100%	100%	100%	100%	100%
Very cost-effective	100%	100%	100%	100%	100%	100%	100%	100%	100%
Cost-effective	100%	100%	100%	100%	100%	100%	100%	100%	100%
Lifetime									
HALYs gained, thousands	155 (137; 174)	100 (85; 117)	78 (69; 89)	230 (203; 259)	461 (407; 518)	64 (56; 72)	155 (137; 174)	155 (137; 174)	155 (137; 174)
Net costs, million USD	-271 (-310; -235)	-169 (-203; -140)	-127 (-150; -109)	-413 (-470; -361)	-311 (-371; -257)	-154 (-175; -134)	-265 (-304; -228)	-277 (-316; -241)	-264 (-303; -228)
ICER, USD per HALY gained[2]	dominant (dominant; dominant)	dominant (dominant; dominant)	dominant (dominant; dominant)	dominant (dominant; dominant)	dominant (dominant; dominant)	dominant (dominant; dominant)	dominant (dominant; dominant)	dominant (dominant; dominant)	dominant (dominant; dominant)
Probabilities									
Cost saving	100%	100%	100%	100%	100%	100%	100%	100%	100%
Very cost-effective	100%	100%	100%	100%	100%	100%	100%	100%	100%
Cost-effective	100%	100%	100%	100%	100%	100%	100%	100%	100%

[1]HALY, health-adjuested life years; ICER, incremental cost-effectiveness ratio; iTFA, industrial trasn-fatty acids; USD, United States Dollars.[2]Dominant ICER indicate a cost-saving intervention (i.e., negative net costs) that improves health (i.e., positive HALYs generated).

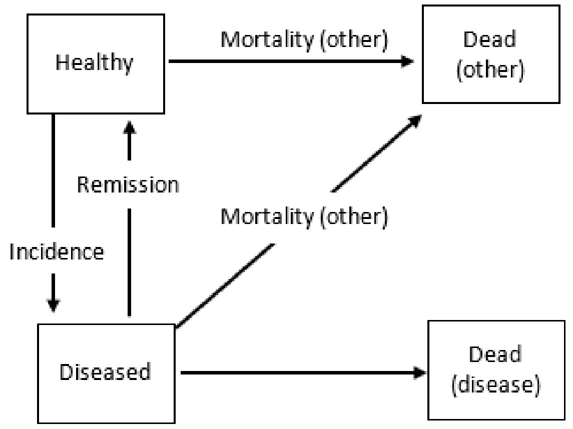

Figure S1: Conceptual model of DisMod II. In this study, 5- year age group and sex specific parameters of IHD incidence, prevalence, and mortality for Kenya were added to DisMod II, which generated internally consistent estimates using background Kenyan population size and all-cause mortality [3].

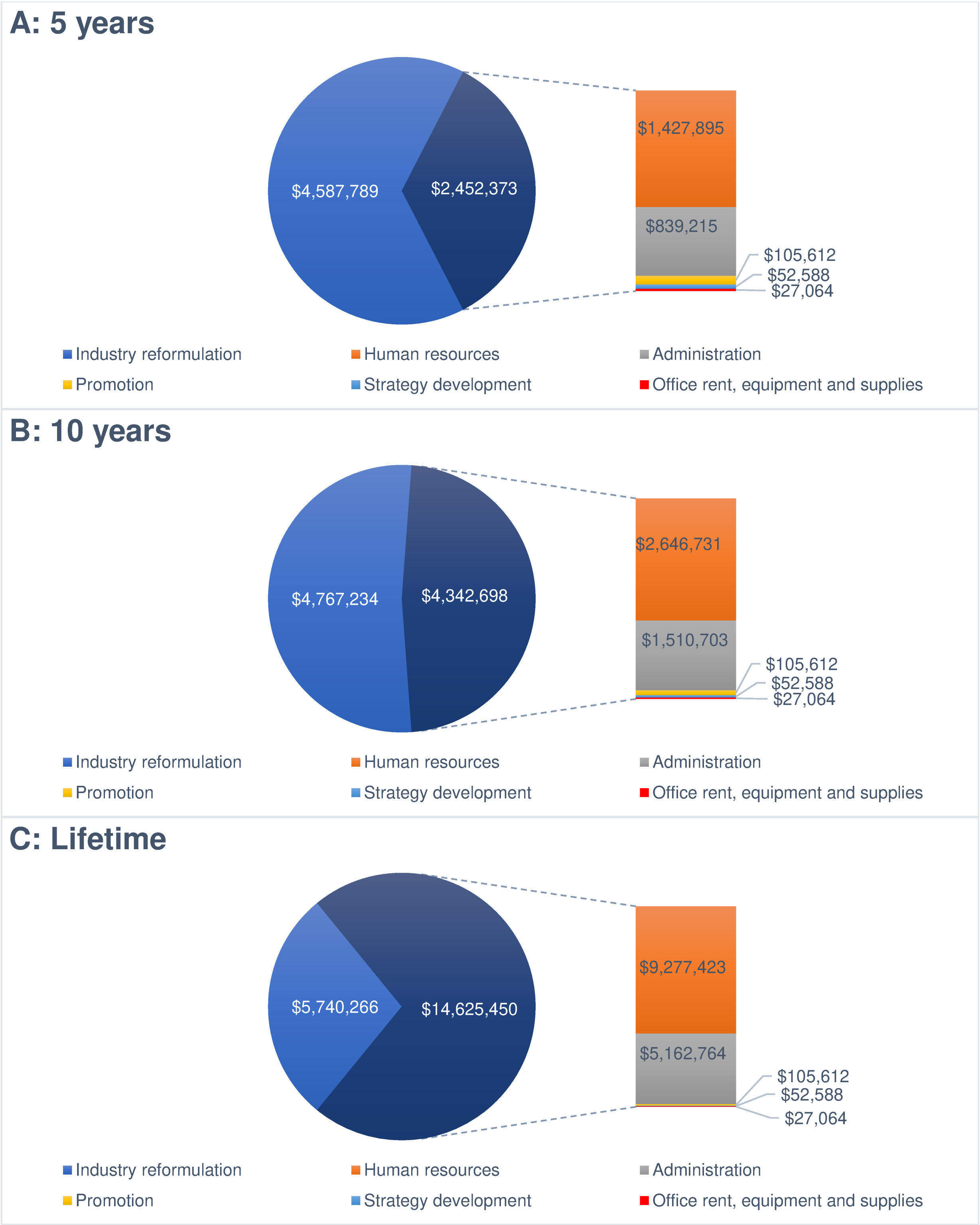

Figure S2. Estimated policy costs of a mandatory limit of industrial trans fatty acids in the Kenyan food chain over 5 years (A), 10 years (B), and the population lifetime (C). Industry costs for product reformulation are depicted in light blue, while total government costs are represented in dark blue, with the individual cost categories presented in the right-hand bar graphs.

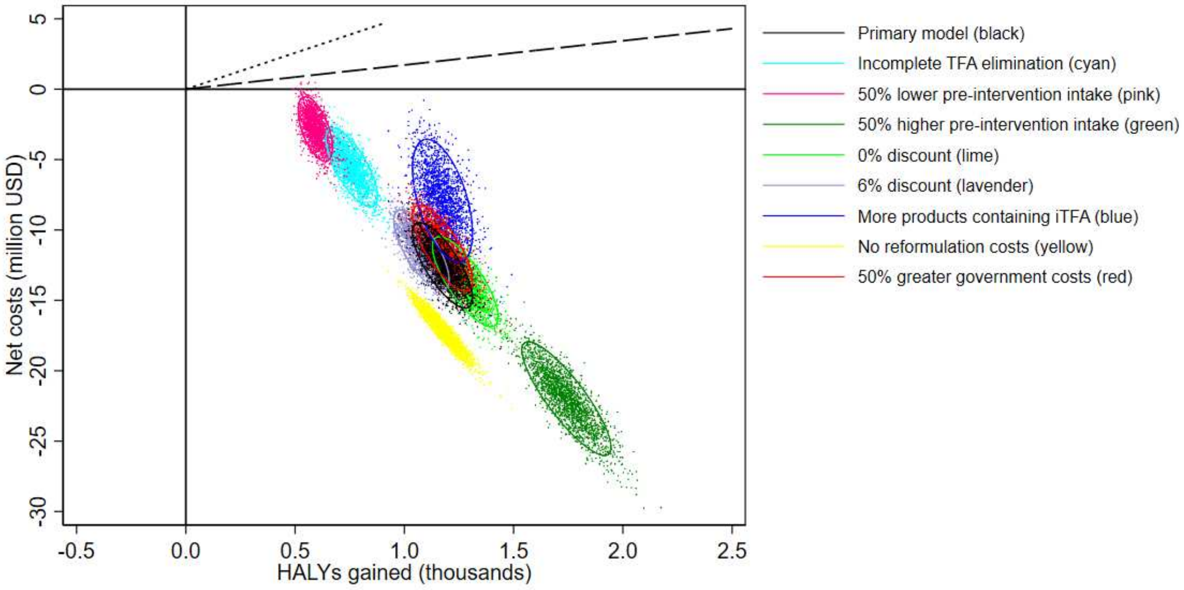

Figure S3. Net costs and HALYs gained during the first 5 years after policy implementation estimated in the primary model and in deterministic sensitivity analyses. Dotted and dashed black lines indicate thresholds for cost-effective and very cost-effective interventions, respectively.
