# Supplementary material for: Estimated health benefits, costs and cost-effectiveness of eliminating dietary industrial trans fatty acids in Kenya: cost-effectiveness analysis
Source: BMJ Glob Health. 2023 Oct 17;8(10):e012692. doi: 10.1136/bmjgh-2023-012692 (PMC10583044; doi:10.1136/bmjgh-2023-012692)
Supplement: Supplementary data [file bmjgh-2023-012692supp002.pdf]

Reflexivity Statement

1. **How does this study address local research and policy priorities?**
   Kenya is considering policy options to regulate industrial trans-fatty acids (iTFA)[11] However, there is currently no limit (voluntary or mandatory) of iTFA content in foods, fats, and oils. Quantitative estimates and corresponding uncertainty of potential health and economic impact and cost-effectiveness of a best-practice mandatory limit on iTFA content (≤2% of all fats) in foods, fats, and oils in Kenya, could inform policy makers considering regulations related to iTFA in Kenya.

2. **How were local researchers involved in study design?**
   A draft of the study proposal was shared with stakeholders from government and local and global NGOs who were invited to a virtual meeting to discuss the objectives and methodology of the study.

3. **How has funding been used to support the local research team?**
   No primary data was collected for this modelling study, and all analyses were conducted by researchers outside of Kenya. No funding for this specific work was allocated to researchers in Kenya.

4. **How are research staff who conducted data collection acknowledged?**
   No primary data was collected for this modelling study, and all analyses were conducted by researchers outside of Kenya.

5. **Do all members of the research partnership have access to study data?**
   All study data is available in the manuscript or the supplementary material. All members of the research partnership have access to the study data.

6. **How was data used to develop analytical skills within the partnership?**
   The research team was divided into smaller groups to identify and prepare input data for the model. The groups met and presented their findings in reoccurring virtual meetings.

7. **How have research partners collaborated in interpreting study data?**
   All members of the research team have provided critical feedback to preliminary results and multiple drafts of the manuscript.

8. **How were research partners supported to develop writing skills?**
   The lead author (MM) prepared the initial draft with support from early career researchers (LNA & MNW) and all members reviewed the drafts critically.

9. **How will research products be shared to address local needs?**
   The findings will be presented in a study report and in an open-access journal article for global dissemination. A dissemination seminar or work shop together with local partners is planned in Kenya to coincide with the open-access publication of the study findings.

10. **How is the leadership, contribution and ownership of this work by LMIC researchers recognised within the authorship?**
    This modelling study was led by researchers outside of Kenya and thus we acknowledge that the authorship team is predominantly based in high-income countries.

11. **How have early career researchers across the partnership been included within the authorship team?**

We have included early career researchers (LNA & MNW) within the authorship team. They have led the identification and preparation of key model inputs, contributed to the study design, drafted parts of the initial draft, and critically reviewed the manuscript before submission. We acknowledge that they are based in high-income countries.

12. **How has gender balance been addressed within the authorship?**
    Four authors are female (MNW, CA, LS, and LKC) and four authors male (MM, LNA, JLV, and JHYW)

13. **How has the project contributed to training of LMIC researchers?**
    The early career researchers (LNA & MNW) are based in a high-income country (Australia).

14. **How has the project contributed to improvements in local infrastructure?**
    This project has not directly contributed to improvements in local infrastructure.

15. **What safeguarding procedures were used to protect local study participants and researchers?**
    No primary data was collected for this modelling study, therefore this question is not directly applicable.
